# Cranial organs at risk delineation: heterogenous practices in radiotherapy planning

**DOI:** 10.1186/s13014-021-01756-y

**Published:** 2021-02-04

**Authors:** Guillaume Vogin, Liza Hettal, Clarisse Bartau, Juliette Thariat, Marie-Virginie Claeys, Guillaume Peyraga, Paul Retif, Ulrike Schick, Delphine Antoni, Zsuzsa Bodgal, Frederic Dhermain, Loic Feuvret

**Affiliations:** 1 Department of Radiation Oncology, Institut de Cancérologie de Lorraine, Vandoeuvre Les Nancy, France; 2 IMoPA, UMR 7365 CNRS-Université de Lorraine, Vandoeuvre Les Nancy, France; 3 Centre National de radiothérapie du Grand-Duché de Luxembourg, Centre François Baclesse, Boîte postale 436, 4005 Esch sur Alzette, Luxembourg; 4 Aquilab SAS, Parc Eurasanté - 250 rue Salvador Allende, Loos, France; 5 Département de Radiothérapie, Centre François Baclesse/ARCHADE, 3 Av General Harris, Caen, France; 6 Laboratoire de Physique Corpusculaire IN2P3/ENSICAEN - UMR6534 - Unicaen, Normandie Université, Caen, France; 7 Institut Daniel Hollard, Service de radiothérapie, Grenoble, France; 8 Service de Radiothérapie, Institut Universitaire du Cancer de Toulouse (Oncopole), Toulouse, France; 9 Service de Radiothérapie, CHR de Metz-Thionville Site Mercy, Metz, France; 10 Département de radiothérapie, CHU de Brest, Brest, France; 11 Département de radiothérapie, Institut de Cancérologie Strasbourg Europe (ICANS), Strasbourg, France; 12 Radiation Oncology Department, Gustave Roussy University Hospital, Villejuif, France; 13 Department of Radiation Oncology, AP-HP, Hôpitaux Universitaires La Pitié Salpêtrière - Charles Foix, Sorbonne Université, Paris, France

**Keywords:** Neuroimaging, Inter individual variability, Segmentation, Continuing education, Radiotherapy

## Abstract

**Background:**

Segmentation is a crucial step in treatment planning that directly impacts dose distribution and optimization. The aim of this study was to evaluate the inter-individual variability of common cranial organs at risk (OAR) delineation in neurooncology practice.

**Methods:**

Anonymized simulation contrast-enhanced CT and MR scans of one patient with a solitary brain metastasis was used for delineation and analysis. Expert professionals from 16 radiotherapy centers involved in brain structures delineation were asked to segment 9 OAR on their own treatment planning system. As reference, two experts in neurooncology, produced a unique consensual contour set according to guidelines. Overlap ratio, Kappa index (KI), volumetric ratio, Commonly Contoured Volume, Supplementary Contoured Volume were evaluated using Artiview™ v 2.8.2—according to occupation, seniority and level of expertise of all participants.

**Results:**

For the most frequently delineated and largest OAR, the mean KI are often good (0.8 for the parotid and the brainstem); however, for the smaller OAR, KI degrade (0.3 for the optic chiasm, 0.5% for the cochlea), with a significant discrimination (*p* < 0.01). The radiation oncologists, members of *Association des Neuro-Oncologue d’Expression Française* society performed better in all indicators compared to non-members (*p* < 0.01). Our exercise was effective in separating the different participating centers with 3 of the reported indicators (*p* < 0.01).

**Conclusion:**

Our study illustrates the heterogeneity in normal structures contouring between professionals. We emphasize the need for cerebral OAR delineation harmonization—that is a major determinant of therapeutic ratio and clinical trials evaluation.

## Background

Radiotherapy (RT) is delivered as definitive treatment or adjuvant following surgical resection in primary or secondary malignant or benign intracranial tumors [1]. However, RT can be followed by late toxicity in 5–10% of the patients with additional societal costs among survivors [2–4]. The most common sequelae include radiation necrosis, neurocognitive effects, cerebrovascular effects, neurosensory deficits, endocrinopathies and radiation-induced brain tumors. Besides dose, one of the determinants of these complications is the volume of normal tissue irradiated [5]. Modern techniques, e.g. stereotactic, intensity-modulated, image-guided or proton-beam RT, may improve the targeted delivery of RT to better protect surrounding tissue by means of a steep dose gradient in the tissues [6]. The organs at risk (OAR) of radiotherapy-associated toxicity, including optic nerves, optic chiasm, retinae, lenses, brainstem, pituitary, cochlea and hippocampus, should be (properly) delineated. This step in the treatment planning is performed manually or (semi) automatically and ultimately validated by the radiation oncologist on the treatment planning system from reference computed tomography (CT) images of the patient acquired in the treatment position before the initiation of the treatment. OAR delineation recommendations have been published—however, in numbers far less important than repositories of tumor segmentation. There are also some interactive atlases marketed online (e.g. https://www.imaios.com/fr/e-Anatomy/Tete-et-cou/Crane-TDM) and atlas-based segmentation software not used in routine due to a poor accuracy, especially for small structures [7]. Segmentation is therefore one of the most crucial steps in treatment planning as dose distribution and optimization directly depend on the accuracy of delineation—especially with the most advanced techniques. Inter-observer variability of tumor target volumes delineation has been emphasized in several locations—including brain—with an impact on tumor control probability [8–11]. However, to our knowledge, inter-observer variability of cephalic OAR delineation has not been formally reported. The aim of this pragmatic study was to evaluate the inter-individual variability of CT-based cranial OAR delineation in neurologic radiation oncology practice between various centers and professionals dedicated to this task.

## Methods

This was a multicenter study endorsed by GRANOCEF, the Radiation Group of the *Association des Neuro-Oncologue d’Expression Française* (ANOCEF).

### Case and procedure

Anonymized contrast-enhanced CT scans in treatment position of one patient treated for a solitary brain metastasis in intent of Stereotactic body radiation therapy was used for delineation and analysis. CT scans encompassing the whole brain were performed according to the following procedure: acquisition extended from the vertex to C7 with 1 mm slices every 1 mm; max 500 slices, FOV: 350 mm, image resolution: 512 × 512 (pixel size: 1.46 pxl/mm), 120 kV, 370 mAs mean (auto modulation). Mean estimated CTDI vol = 45 mGy; iodinated contrast medium injected 10 min prior acquisition at the concentration of 1.5 mL/kg—maxi 100 mL.

All images were then transferred to Isogray™ treatment planning system (Dosisoft, Cachan, France) in DICOM format. According to published international recommendations, two referent senior radiation oncologists expert in neuro-oncology (8 and 15-year seniority, members of ANOCEF) produced a unique consensual contour set for the following 9 OAR: left parotid, left optic nerve (LON), optic chiasm, brainstem, pituitary, left cochlea, left internal acoustic meatus (LIAM), left hippocampus and anterior segment of the left eyeball (ASLE) [12–16]. We uploaded the anonymized CT scan along with the “expert” contours to the Aquilab Share Place™ platform (Aquilab, Lille, France). The associated diagnostic MR examination was sent separately to the participating centers and image fusion/registration was optionally performed on site secondarily.

### Population studied

In France notably, OAR delineation can be delegated to other professionals under the supervision of the radiation oncologist. Therefore, we solicited the professionals involved in cranial OAR delineation in their daily activity in eight RT centers in north-eastern France—academic and private. Professionals could be radiotherapy technologists (RTT) or dosimetrists, residents or senior radiation oncologists (RO). These so called “observers” were stratified according to their seniority: less than 3 years, between 3 and 10 years and more than 10 years of practice.

In addition, in December 2017, we solicited the expert radiation oncologists involved in ANOCEF (Association of French-speaking Neuro-oncologists) in France as well as those participating in our *European Greater Region Radiation Oncology Consortium* (Universitätsklinikum des Saarlandes, Homburg/Saar—Germany, Centre François Baclesse—Centre National de Radiothérapie du Grand-Duché de Luxembourg, Esch s/Alzette—Luxembourg, Centre Hospitalier Universitaire de Liège, Liège—Belgium).

### Centralized analyze interface

AQUILAB made available a dedicated secured website to download the pre-mentioned anonymized DICOM imaging as well as a procedure to delineate the set of OAR (procedure available upon request) and upload the contours. Each participant had to register with personal ID and password prior to the exercise. The participants (observers) delineated the set of OAR, on all slices, blind to other contours, using their own segmentation tools and according to their daily practice. The resulting set of OAR was labeled with an anonymous ID. In parallel, we collected the occupation, seniority and level of expertise in the position of all participants.

### Contours comparison method

The variability and the differences in the delineated volumes were quantified using Artiview™ v2.8.2 software (Aquilab, Loos Les Lille, France).

The comparative analysis was performed by using specific metrics (Additional file 1: Figure S1) [17–21]. The standard deviation was calculated.

If Cn refers to the contour determined by the observer n and CR the reference contour, then these two contours can be compared using the following criteria:

Overlap ratio (OV) is defined as the ratio between the intersection of Cn with CR and their union [22]. (Optimal value = 1)

$$OV = \frac{{{\text{Cn }} \cap {\text{CR}}}}{{{\text{Cn }} \cup {\text{CR}}}}$$

Dice similarity coefficient or Kappa Index (KI) or Cohen’s Kappa was used to determine the agreement between the reference contour and the user’s contour, as described elsewhere [23]. Kappa values of 0.81 to 1.0 indicate excellent agreement, 0.61 to 0.80 good agreement, 0.41 to 0.60 moderate agreement, and ≤ 0.40 poor agreement.

From Receiver Operating Characteristics (ROC) we can also measure the following indexes:

- The volumetric ratio (VR) defined as the ratio between Cn and CR; (Optimal value = 1)
  $$VR = \frac{{{\text{Cn}}}}{{{\text{CR}}}}$$
- The Commonly Contoured Volume (VCC) is defined as the ratio between the intersection of Cn with CR and CR; (Optimal value = 1)
  $$VCC = \frac{{{\text{Cn }} \cap {\text{CR}}}}{{{\text{CR}}}}$$
- The Supplementary Contoured Volume (SCV) is defined as the ratio between the difference of Cn with $\overline{{{\text{CR}}}}$ (defining the outside of the reference contour) and Cn (Optimal value = 0)
  $$VSC = \frac{{{\text{Cn}} \cap \overline{{{\text{CR}}}} }}{{{\text{Cn}}}}$$

### Taking into account the inter-individual variability linked to the software

In order to study the human factor separately, it appeared necessary to evaluate three types of contouring uncertainties that could be linked to the technology with a second study: (1) inaccuracy of the manual contouring tools of the TPS used by the observers, (2) imprecision of the thresholding automatic when used to contour, (3) uncertainties related to the import–export of structures from the local TPS to the centralized analyze interface.

To address these three points, the experts delineated the contralateral (right) structures. AQUILAB then sent back the observers the reference contrast-enhanced CT scans as well as a RT-STRUCT file containing the right OARs and additional structures to be contoured. One radiation oncologist per center had then to carry out 2 additional tasks, ideally using the same station as during the first exercise.

1. Contour manually with its own tools 4 imposed left structures made hyperdense: left parotid, LON, left hippocampus and L-ASLE.
2. Contour these same 4 left structures with automatic thresholding (3000 HU).

The RT-STRUCT files enriched with the contours produced were then centralized back for analysis with the previously described metrics.

### Statistical analysis

A descriptive analysis of original contours was performed on all variables. Quantiles, mean and standard deviation were evaluated for quantitative variables. Qualitative variables were summarized with their levels’ frequencies. For statistical purposes, centers with only one professional were pooled within center #6. Comparisons between different groups was assessed by a one-way analysis of variance (ANOVA). When a significant difference was found, we used a Tukey’s post-hoc test, which was corrected for multiple comparison, to perform individual comparisons.

In the second study, to assess the three potential technical interferences a Mann–Whitney test was used. Comparisons of organ delineation performances for each exercise were assessed by a Kruskal–Wallis test. When a significant difference was found, we used a Mann–Whitney test, which was corrected for multiple comparison, to perform individual comparisons.

Significance threshold was set to *p* < 0.05. All statistical analyses were performed on R version 3.5.3 (March 11th, 2019).

## Results

The database was frozen on April 2018.

Professionals from sixteen centers uploaded their sets of structures representing overall 57 professionals: 33 senior radiation oncologists (including 11 ANOCEF members), 18 residents training in radiation oncology (out of whom one—AQ21—was excluded due to an aberrant contour set) and 6 “specialized” RTT (Table 1). Two datasets were not exploitable (AQ23 and 29) as they did not refer to the right exam and therefore appeared shifted. Eight centers were represented by more than 2 professionals and have been analyzed for their own account.

**Table 1:** Characteristics of the observers according to their center, occupation, seniority and expertise level

ID	Center	Occupation	Experience	ID	Center	Occupation	Experience
AQ1	1	RTT	> 10 years	AQ30	6	RO	Fellow (0–2 years)
AQ2	1	RO, ANOCEF	> 10 years	AQ31	6	RO, ANOCEF	3–10 years
AQ3	1	RTT	> 10 years	AQ32	7	RO	> 10 years
AQ4	1	Resident	3rd year	AQ33	7	RO, ANOCEF	> 10 years
AQ5	2	RO	3–10 years	AQ34	7	RO	3–10 years
AQ6	1	RO	3–10 years	AQ35	7	RO	4 years
AQ7	1	RTT	> 10 years	AQ36	7	RO	Fellow (0–2 years)
AQ8	1	RTT	3–10 years	AQ37	7	RO	Fellow (0–2 years)
AQ9	1	Resident	1st year	AQ38	7	RO	Fellow (0–2 years)
AQ10	1	RTT	3–10 years	AQ39	7	Resident	3rd year
AQ11	1	RTT	> 10 years	AQ40	7	Resident	1st year
AQ12	1	Resident	2nd year	AQ41	8	RO, ANOCEF	> 10 years
AQ13	1	RO	3–10 years	AQ42	8	Resident	1st year
AQ14	1	Resident	1st year	AQ43	8	Resident	2nd year
AQ15	3	RO	> 10 years	AQ44	8	RO	3–10 years
AQ16	3	RO	3–10 years	AQ45	8	Resident	1st year
AQ17	3	RO	> 10 years	AQ46	8	Resident	2nd year
AQ18	4	RO	3–10 years	AQ47	8	RO	Fellow (0–2 years)
AQ19	4	Resident	3rd year	AQ48	8	RO	3–10 years
AQ20	4	Resident	3rd year	AQ49	8	Resident	1st year
AQ21	4	Resident	4th year	AQ50	8	Resident	1st year
AQ22	2	RO	> 10 years	AQ51	6	RO, ANOCEF	> 10 years
AQ23	6	RO	fellow (0–2 years)	AQ52	6	RO, ANOCEF	3–10 years
AQ24	5	Resident	1st year	AQ53	6	RO, ANOCEF	Fellow (0–2 years)
AQ25	5	RO	> 10 years	AQ54	6	RO, ANOCEF	> 10 years
AQ26	5	Resident	2nd year	AQ55	9	RO, ANOCEF	Fellow (0–2 years)
AQ27	5	RO	3–10 years	AQ56	6	RO, ANOCEF	> 10 years
AQ28	5	Resident	2nd year	AQ57	9	RO, ANOCEF	> 10 years
AQ29	6	RO, ANOCEF	> 10 years				

*RO* radiation oncologists, *RTT* radiotherapy technologists, *ANOCEF* members of ANOCEF society

### Overall description

Table 2 shows the overall inter-individual performance range in delineation of the 9 OAR. There is a wide dispersion of volume of all OAR—most of the contours being larger than the ones of the experts, except for brainstem and parotid.

**Table 2:** Overall inter-observer contour comparison metrics

	Optimal value	L_parotid	L_ON	Optic chiasm	Brainstem	Pituitary	L_cochlea	LIAM	L_hippocampus	ASLE
Experts										
Volume (cc)	NA	**38.2**	0.5	0.3	**29.2**	0.2	0.1	0.3	2.1	0.9
# slices contoured	NA	**25**	4	2	**20**	3	3	2	8	5
Observers										
n	NA	**53**	54	53	**55**	55	47	54	38	51
Mean volume (cc) (SD)	NA	**32.4 (6.4)**	1.2 (0.5)	1.1 (0.5)	**24.9 (4.2)**	0.4 (0.2)	0.2 (0.1)	0.9 (4.1)	3.4 (2.0)	2.1 (1.6)
Mean # slices contoured (SD)	NA	**21.4 (3.5)**	3.9 (1.3)	2.9 (1.5)	**17.7 (3.2)**	2.5 (0.6)	1.9 (1.0)	2.1 (2.0)	7.8 (1.9)	5.9 (1.8)
Observers versus expert										
Mean VR (SD)	1.0	**0.9 (0.2)**	2.4 (1.0)	3.2 (1.6)	**0.9 (0.2)**	1.7 (0.8)	1.8 (1.3)	3.4 (15.1)	1.6 (1.0)	2.3 (1.8)
Mean VCC (SD)	1	**0.76 (0.15)**	0.69 (0.13)	0.56 (0.23)	**0.74 (0.14)**	0.74 (0.24)	0.65 (0.22)	0.56 (0.31)	0.45 (0.29)	0.82 (0.18)
Mean VSC (SD)	0	**0.11 (0.05)**	0.67 (0.13)	0.77 (0.17)	**0.15 (0.06)**	0.50 (0.21)	0.48 (0.26)	0.41 (0.28)	0.70 (0.21)	0.46 (0.30)
Mean OV (SD)	1.0	**0.7 (0.1)**	0.3 (0.1)	0.2 (0.2)	**0.7 (0.1)**	0.4 (0.2)	0.4 (0.2)	0.4 (0.2)	0.2 (0.2)	0.4 (0.2)
Mean KI (SD)	1.0	**0.8 (0.1)**	0.4 (0.1)	0.3 (0.2)	**0.8 (0.1)**	0.6 (0.2)	0.5 (0.2)	0.5 (0.2)	0.3 (0.2)	0.6 (0.2)
Global OV	1.0	**0.1**	0.0	0.0	**0.1**	0.0	0.0	0.0	0.0	0.0
Global KI	1.0	**0.2**	0.1	0.0	**0.3**	0.0	0.0	0.0	0.0	0.1

Bold values refer to so-called voluminous OAR

*SD* standard deviation, *L_ON* left optic nerve, *LIAM* left Internal acoustic meatus, *ASLE* anterior segment of the left eyeball, *VR* volume ratio, *VCC* volume commonly contoured, *VSC* volume supplementary contoured, *OV* overlap ratio, *KI* Kappa index

**Fig. 1:**
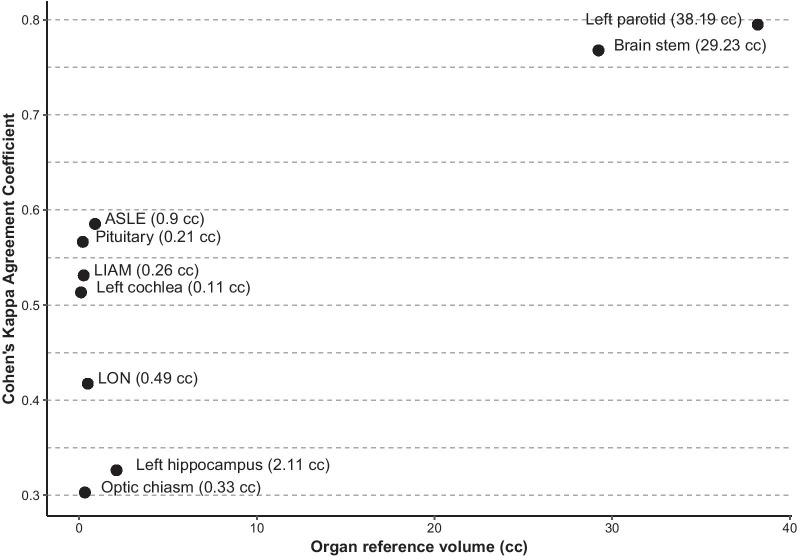
Kappa index values as a function of the mean OAR volume according to the experts

For the most frequently delineated and largest OAR, the mean KI are often good (0.8 for the parotid and the brainstem); however, for the smaller OAR, KI degrade (0.3 for the optic chiasm, 0.5% for the cochlea) (Fig. 1).

**Fig. 2:**
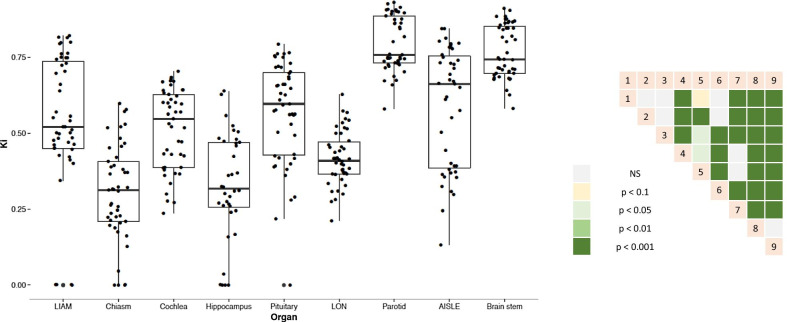
Kappa index values as a function of the OAR (left panel) and inter comparisons (right panel) 1-left cochlea, 2-pituitary, 3-LIAM, 4-optic chiasm, 5-LON, 6-ASLE, 7-left hippocampus, 8-brainstem, 9-left parotid

**Fig. 3:**
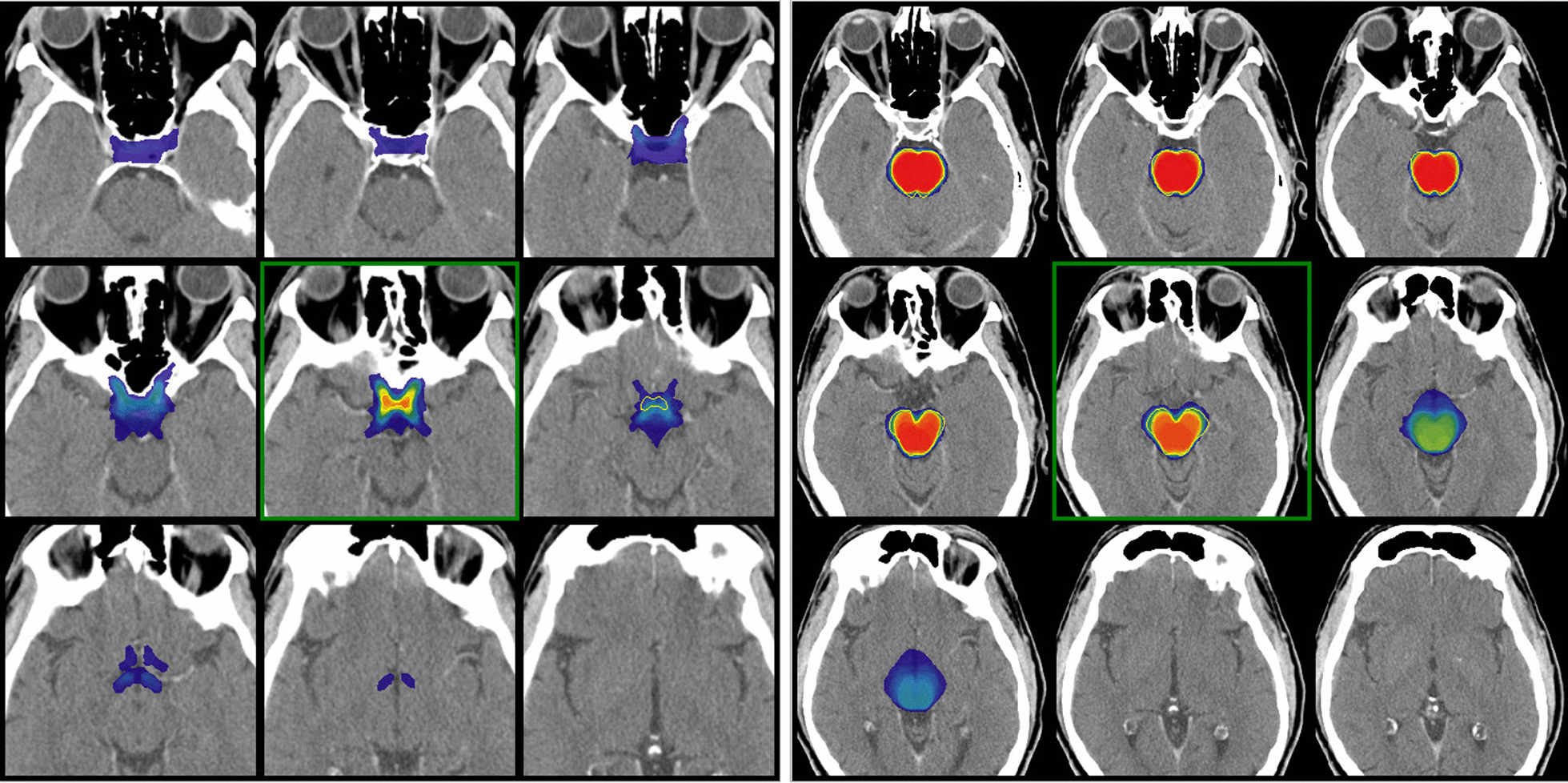
Superposition of the observers’ on the expert (yellow contour) volumes and illustration of the VCC (color wash): left panel: optic chiasm; right panel: brainstem

We could confirm this impact of the OAR volume on the following indicators: VCC (*p* < 2.0 × 10^–16^), VSC (*p* < 2.0 × 10^–16^) and KI (*p* < 2.0 × 10^–16^). The VCC, OV and KI of the largest organs (i.e. parotid and brainstem) were significantly greater than those of the smaller structures—i.e. Chiasm, Cochlea, Pituitary, LON, LIAM, ASLE. (*p* = 1.0 × 10^–7^, *p* < 2.2 × 10^–16^, and *p* < 2.2 × 10^–16^ respectively) (Fig. 2). The VSC and RV of the large organs appeared significantly lower than those of the small organs, respectively *p* < 2.2 × 10^–16^ and *p* = 2.8 × 10^–6^ (Fig. 3).

The hippocampus is rarely well-delineated, especially by non-radiation oncologists (mean KI = 0.3).

### Interclass variations in delineation of the 9 OAR

#### Occupation as comparator

All the RTT who participated in the study represented only one center, so we analyzed only the RO and residents’ populations.

There is a significant difference in the VCC between occupations (*p* = 2.0 × 10^–5^). The RO performed better than the residents (0.68, CI_95%_ = [0.34,1] vs 0.60, CI_95%_ = [0.19,1] respectively, *p* = 0.012). There is no significant difference for any of the other performance parameters studied.

#### Seniority as comparator

We did not find any significant difference in the performance parameters studied based on the experience of the subjects.

#### ANOCEF membership as comparator (physicians only)

The RO members of ANOCEF society performed better in all indicators compared to RO non-members (*p* < 0.01 except for VCC with *p* = 0.06) (Additional file 2: Figure S2).

#### Center as comparator

**Fig. 4:**
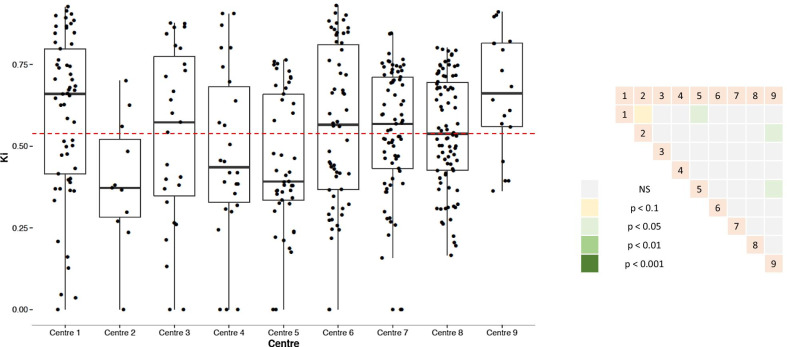
KI according to the center (all professionals included) (left panel) and inter comparisons (right panel). The intracentric variance is reflected by the height of the box plot. Center 6 brings together all the centers that provided only one experimenter

Our exercise was effective in separating the different participating centers with 3 of the reported indicators. Indeed, we noted a significant difference for the OV (*p* = 4.1 × 10^–4^), the VCC (*p* = 3.1 × 10^–8^), and the KI (*p* = 6.1 × 10^–4^) and a trend for the VSC (*p* = 1.5 × 10^–2^) (Fig. 4). Noteworthily centers 1 and 9 stand out from their peers, in particular by superior OV and VCC.

### Weight of technical inaccuracies on the results

Seven radiation oncologists from 7 centers took part to the second part of the study assessing software impact on delineation inaccuracy. In Tables 3 and 4, we report and intercompare the mean KI for the three different endpoints assessed with the same endpoint for the original structures primarily evaluated. The variability linked to the human factor (inter-observer) is statistically superior to the technical variability, regardless of the organ.

**Table 3:** mean KI (standard deviation) for the different endpoints assessed in the two studies

	Export/import effect (right OAR)	Automatic segmentation of imposed structures with imposed thresholding (left OAR)	Manual segmentation of imposed structures (left OAR)	Original structure (left OAR)
Parotid	0.989 (0.070)	0.969 (0.029)	0.964 (0.018)	0.865 (0.106)
ON	0.975 (0.017)	0.764 (0.249)	0.841 (0.082)	0.569 (0.233)
Hippocampus	0.989 (0.007)	0.920 (0.085)	0.926 (0.036)	0.593 (0.322)
ASLE	0.987 (0.009)	0.895 (0.112)	0.908 (0.049)	0.716 (0.231)

**Table 4:** Intercomparaison of the three technical parameters with the original manual delineation (*p* values from a Mann–Whitney test)

	Original structures versus export/import effect	Original structures versus manual segmentation of imposed structures	Original structures versus automatic segmentation of imposed structures with imposed thresholding
Parotid	0.000480113	0.00016129	0.001558491
ON	0.000461476	1.23E−06	0.000666839
Hippocampus	0.000664306	5.31E−06	0.00325495
ASLE	0.000504096	1.81E−06	0.003649961

However, the magnitude of technical inaccuracy could be organ dependent—according to the typology of uncertainty. After automatic contouring with thresholding, the conformity of the contours to the reference was identical, whatever the structure. In the case of manual delineation of a defined structure, the KI was maximum for the parotid and minimum for the ON. There was a mostly imperceptible import–export effect depending on the organ (Additional file 3: Table S1).

## Discussion

After demonstrating the superiority of human factor over technical factors on interobserver delineation variability, our study illustrates the heterogeneity in normal structures contouring between professionals. Compared to international guidelines—sometimes slightly different [13, 24, 25]—observers tended to overestimate the volume of most OAR and small OAR in particular. This is especially significant for the optic structures. Depending on the thickness of the slices, these structures are frequently difficult to identify, including on MRI, sometimes with a shift due to the inaccuracy of the fusion. This could impact PTV coverage for tumors located close by. Moreover, the spatial overlap of these structures between experts and observers appears weak, which may expose them to overdosage and toxicity. On the contrary, at similar volume, the auditory structures had a volume comparable to that of the experts with a low dispersion and a correct agreement (Kappa index > 0.5). Observers have well integrated the value of the bone window for the accuracy of cochlea delineation. Larger OARs such as the brainstem and parotid were under-evaluated but without impact on the Kappa index. There were frequently inter-observer variations on the cranio-caudal length of these structures and therefore on the number of contoured sections. We could observe a correlation between OAR volume and agreement coefficient as expected [7].

In a more analytical way, we could not evidence any impact of occupation or seniority on delineation agreement. ANOCEF membership was nonetheless associated with the best delineation performance. These practitioners may be involved in quality assurance of clinical trials in neuro-oncology. It was difficult to compare individuals within the same occupation because we wanted to offer the exercise only to staff who were experienced in brain radiologic anatomy. Noteworthy, only one center routinely delegates the delineation of brain OAR to the RTT, which is provided essentially by the physicians in the other centers. Other facilities may involve medical physicists. Finally, we proposed the exercise only once and we cannot present intra-individual variability in OAR segmentation since it was not our objective.

The objective of this work was to evaluate the participants’ abilities to recognize and draw the OARs in CT brain imaging. Observers have frequently deliberately omitted to draw the hippocampus. Hippocampus delineation performance was not interpretable here as it was the only OAR that needed to be segmented on the MRI [26]. The out-room MRI that had initially been fused for delineation included thicker slices than the planning CT in the plane of acquisition and was misleading, as the slices had averaged the abnormal signal over the full thickness of the slice and partial voluming was observed [27]. We believe that the added value of MRI for contouring the majority of OAR is not major—especially with millimeter-thick CT sections. The use of image fusion is moreover associated with a risk of geometric inaccuracy as a result of the fusion process when performed individually in each participating center, as well as workflow changes [28].

Although very time-consuming and repetitive, OAR delineation should not be neglected; multiple risks are described in relation to the nature of software tools and especially the contrast thresholds used, the number of segmented sections, the extraction or automated 3D expansion method of the contours, or the quality of image fusion when used. Most contouring solutions available on the market are incorporated into the treatment planning systems and apply image-processing capabilities to better distinguish a structure of the rest of the image. Misuse of these tools can thus generate a degradation of the geometric accuracy of the dose distribution in the patient, leading ultimately to possible under-dosage in the periphery of the tumor and a possible over-dosage in the neighboring OAR without any apparent gap at the prescription point [29]. The human factor is certainly even more important. OAR segmentation is directly based on anatomy and radiologic anatomy knowledge in addition to the proper use of contouring tools. The precision of OAR segmentation can thus have a major impact on the therapeutic ratio as it has been shown for tumor volumes [8–10]. According to the Quality Assurance of EORTC randomized trials in neuro-oncology, a significant proportion of the major deviations recorded were attributable to improper OAR delineation [30, 31].

Based on the results of this study, we estimate that the delegation of delineation presumes a customized or generic training in radiologic anatomy scheduled more or less formally in each department. It may also be considered to regularly recertify the professionals to the delineation of OAR as part of continuing education. More broadly, harmonizing the delineation of OAR will certainly help the community to standardize practices and improve the robustness of the results of clinical trials and refine the knowledge of dose–response relationships for OAR.

Automatic recognition of anatomical structures without human intervention is a trendy topic [7, 32]. To date, no (semi) automated segmentation tool is routinely implemented. The prospects for (semi) automatic contouring are attractive due to the reduced inter/intra-observer variability and the time saved on the workflow. However, contrarily to the software, man is able to analyze the unexpected variations in anatomy. The software can only repeat what has been encoded into it. It seems thus essential to us that radiotherapy professionals understand physiological radioanatomy as a major determinant of therapeutic outcomes.

## Conclusion

Delineation of OAR is a critical step in radiation planning. We demonstrated the deviation compared to international guidelines, especially for smaller structures; belonging to a neuro-oncology society is a protective factor. Even if OAR contouring is being automated in a more or less near future, it seems essential to harmonize practices in order to (1) avoid deviations from the treatment plan at the individual level, and (2) collectively not introduce bias in the results of radiotherapy clinical trials.

## Supplementary Information

**Additional file 1: Figure S1.** Surface metrics used in the study. CR: Reference contour delineated by the experts according to published recommendations (red disk); Cn: Observer(s) contour(s) (blue disk).

**Additional file 2: Figure S2.** KI according to ANOCEF membership, **p < 0.01.

**Additional file 3: Table S1.** Investigation of organ effect on the technical uncertainties (p values); shaded cells = lack of statistical significance.

## Data Availability

The datasets used and/or analysed during the current study are available from the corresponding author on reasonable request.

## Abbreviations

RT: Radiotherapy
OAR: Organ(s) at risk
RTT: Radiotherapy technologist(s)
RO: Radiation oncologist(s)
KI: Kappa index
