# Supplementary figures and images for: Cranial organs at risk delineation: heterogenous practices in radiotherapy planning
Source: Radiat Oncol. 2021 Feb 4;16:26. doi: 10.1186/s13014-021-01756-y (PMC7863275; doi:10.1186/s13014-021-01756-y)

## Slide 1
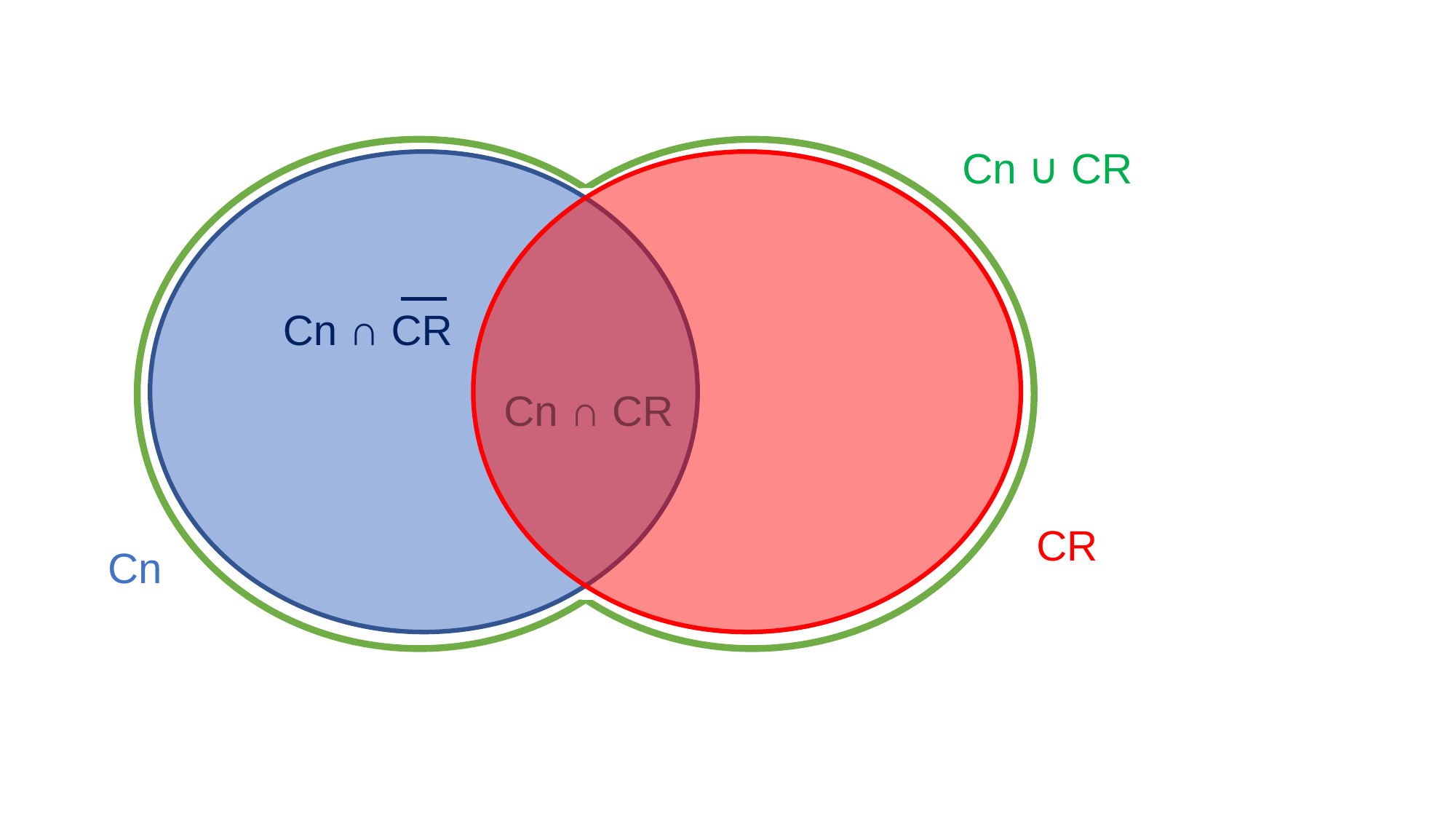

Cn ∪ CR
Cn ∩ CR
Cn ∩ CR
CR
Cn

Supplement: Supplementary file 1 — Additional file 1: Figure S1. Surface metrics used in the study. CR: Reference contour delineated by the experts according to published recommendations (red disk); Cn: Observer(s) contour(s) (blue disk). [file 13014_2021_1756_MOESM1_ESM.pptx]

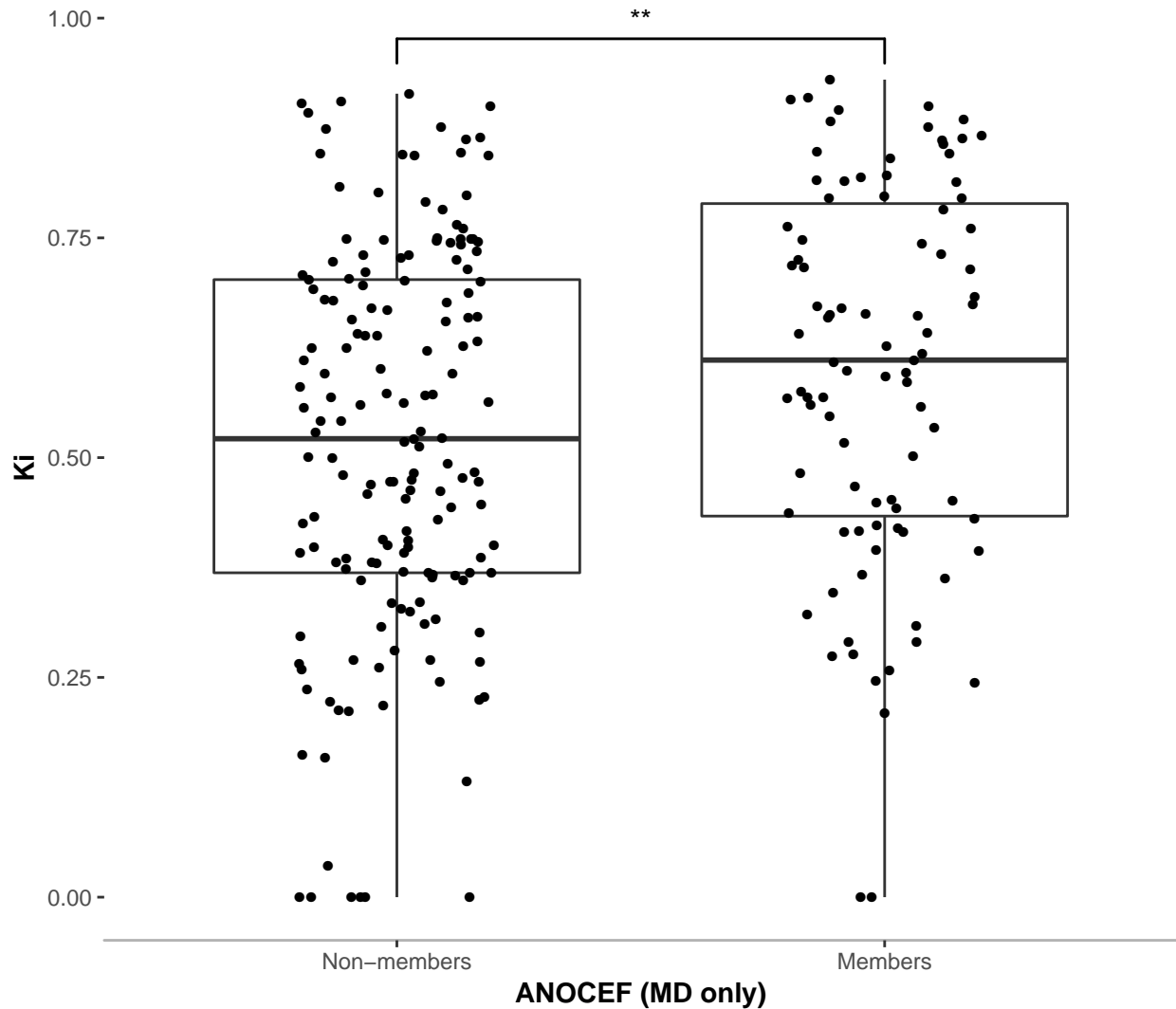

Supplement: Supplementary file 2 — Additional file 2: Figure S2. KI according to ANOCEF membership, **p < 0.01. [file 13014_2021_1756_MOESM2_ESM.pdf]
