# Supplementary material for: Cranial organs at risk delineation: heterogenous practices in radiotherapy planning
Source: Radiat Oncol. 2021 Feb 4;16:26. doi: 10.1186/s13014-021-01756-y (PMC7863275; doi:10.1186/s13014-021-01756-y)
Supplement: Supplementary file 3 — Additional file 3: Table S1. Investigation of organ effect on the technical uncertainties (p values); shaded cells = lack of statistical significance. [file 13014_2021_1756_MOESM3_ESM.docx]

Supplementary Table 1

|  | **Parotid vs Hippocampus vs ON vs ASLE** | **Parotid vs ON** | **Parotid vs Hippocampus** | **Parotid vs ASLE** | **ON vs Hippocampus** | **ON vs ASLE** | **Hippocampus vs ASLE** |
| --- | --- | --- | --- | --- | --- | --- | --- |
| **Manual segmentation of imposed structures (left OAR)** | 0.000308433 | 0.004329004 | 0.004329004 | 0.004329004 | 0.006493506 | 0.018181818 | 0.17965368 |
| **Automatic segmentation of imposed structures with imposed thresholding (left OAR)** | 0.30842 | NA | | | | | |
| **Export/import effect (right OAR)** | 0.003301495 | 0.02035202 | 0.02035202 | 0.02035202 | 0.08090541 | 0.23736861 | 0.23736861 |
| *Statistical method used* | *Kruskal Wallis test* | *Mann-Whitney test with Benjamini et Hoechberg corrections for multiple comparisons* | | | | | |
